# The first observation of osmotically neutral sodium accumulation in the myocardial interstitium

**DOI:** 10.1038/s41598-021-01443-8

**Published:** 2021-11-11

**Authors:** I. Artyukov, G. Arutyunov, M. Bobrov, I. Bukreeva, A. Cedola, D. Dragunov, R. Feshchenko, M. Fratini, V. Mitrokhin, A. Sokolova, A. Vinogradov, A. Gianoncelli

**Affiliations:** 1 P.N.Lebedev Physical Institute RAS, 53 Leninsky Prospekt, Moscow, 119991 Russia; 2 Pirogov Russian National Research Medical University, 1 Ostrovitianov St., Moscow, 117997 Russia; 3 Moscow Regional Research and Clinical Institute (MONIKI), 61/2 Shchepkina St., Moscow, 129110 Russia; 4 CNR-Institute of Nanotechnology, 5 Piazzale Aldo Moro, 00185 Rome, Italy; 5 Elettra-Sincrotrone Trieste, S.S. 14, km 163.5 in Area Science Park, 34149 Basovizza-Trieste, Italy

**Keywords:** X-rays, Imaging, Fluorescence imaging, Fluorescence spectroscopy

## Abstract

The aim of this study was the detection and quantification of the Na^+^ depositions in the extracellular matrix of myocardial tissue, which are suggested to be bound by negatively charged glycosaminoglycan (GAG) structures. The presented experimental results are based on high resolution X-ray fluorescence (XRF) spectromicroscopy technique used to perform a comparative analysis of sodium containment in intracellular and interstitial spaces of cardiac tissues taken from animals selected by low and high sodium intake rates. The experimental results obtained show that high sodium daily intake can result in a remarkable increase of sodium content in the myocardial interstitium.

## Introduction

The sodium homeostasis processes are traditionally the focus of various studies and experiments^1–3^. Thus, there is a large amount of evidence showing the negative impact of sodium overloading on myocardia function in the form of its lowered diastolic function (see, for example^1^). However, recent studies indicate that homeostasis of sodium is much more complex than previously thought: it includes not only osmotic intracellular and extracellular compartments but also a compartment of the interstitial space in which bound Na^+^ is stored without commensurate water accumulation^4–6^ (“three-compartment model”).

As early as in the twentieth century it has been hypothesized that negatively charged glycosaminoglycans (GAGs) of the extracellular matrix may bind to positively charged cations^7,8^. Thus, sodium becomes osmotically neutral and does not affect the values of arterial pressure (AP) and extracellular fluid volume. We should mention also a process was found to involve the macrophages in response to excessive concentration of sodium in GAG^9^.

In 2003 the first experimental proof of water independent sodium storage was delivered by Titze et al*.* for rat skins^5,6^. In 2007–2011 in the framework of the MARS-500 program^10^, a prolonged and accurate study on human sodium intake/excretion ratio has discovered that amounts of excreted sodium are not related directly to the sodium intake and arterial pressure values. This fact was explained by the existence of osmotically passive sodium deposits in skin and muscle tissues that have been formed earlier under conditions of high sodium diet and made their contribution to the elevated level of sodium in the urine.

To confirm an occurrence of sodium depositions inside striated muscles Kopp et al*.*^11,12^ and Hammon et al*.*^13^ used ^23^Na Magnetic Resonance Imaging (MRI) for studying skin and muscles of the lower legs. Additionally, several MRI studies leading to the conclusion, that the heart is a part of the body’s sodium storage (see, for example^14^) and showing a link between sodium intake and left ventricular hypertrophy^15^.

Our previous investigations with the help of acoustical and X-ray fluorescence analysis (BRUKER S8 Tiger 1 kW WDXRF spectrometer) revealed a correlation between elevated sodium content in heart muscles and arterial hypertension as well as patient’s age^16^. We found out also evidence of sodium-related modification of muscle elasticity of myocardia and related diastolic function.

However, all the previous investigations on sodium accumulation in muscle tissues made with the help of MRI or XRF spectrometers were not able to localize the elevated sodium concentration with an adequate high spatial resolution at the cell level. None of the above-mentioned studies could differentiate between extra and intracellular sodium.

The aim of the presented paper was to detect and to estimate the relative content of sodium depositions in the extracellular matrix of myocardial tissues of rats with the reference to sodium consumption rates.

## Results

The samples of cardiac muscle tissues of rats were studied by means of 3 techniques: histology analysis, synchrotron radiation based X-ray transmission microscopy (STXM) and X-ray fluorescent microscopy (XFM) using the same spatial resolution of about 1 μm.

**Figure 1:**
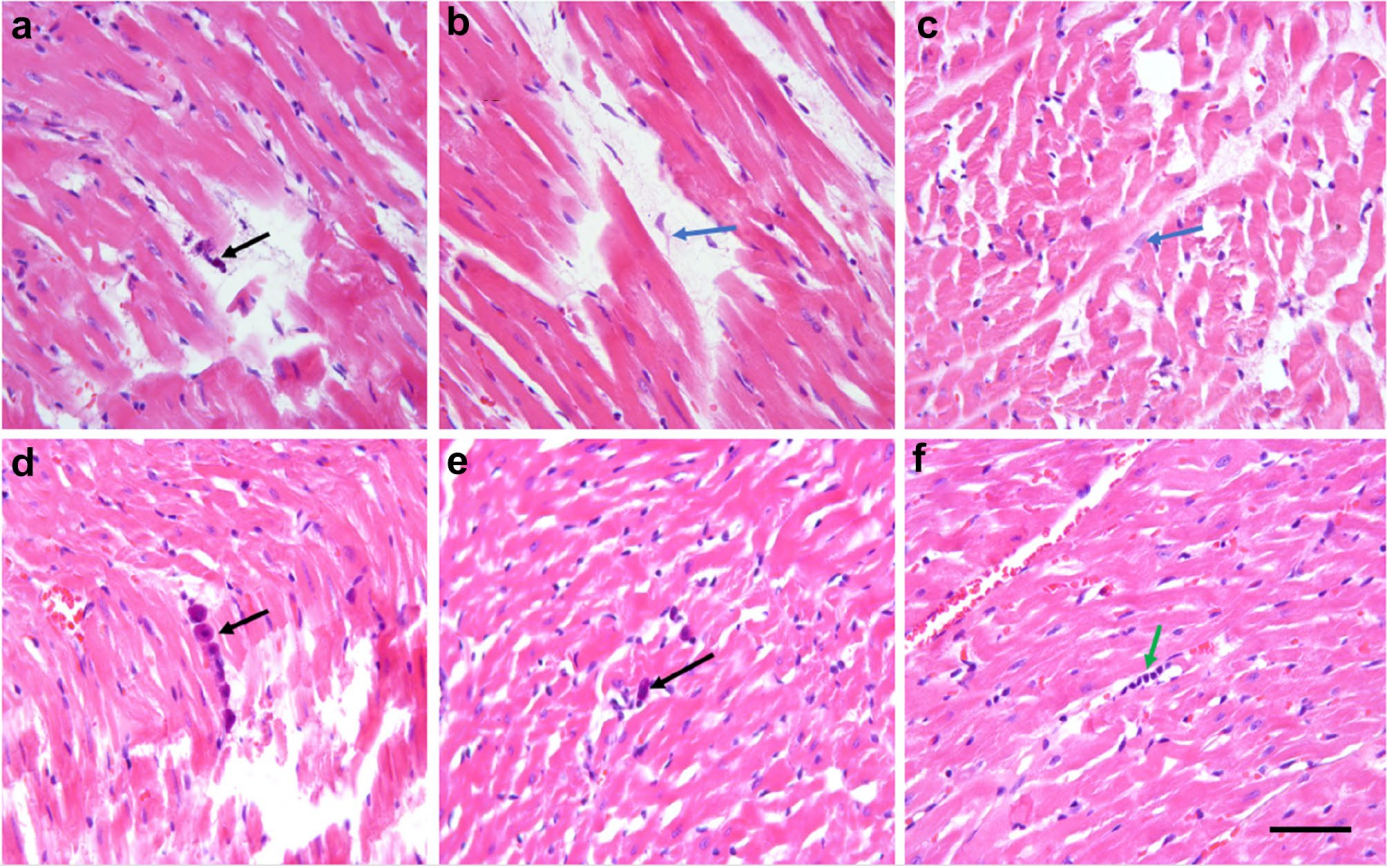
Optical microscopy images of anomalies in heart tissues of rats from high sodium diet group (images **a**–**c**) in comparison with tissues of control animals (images **d**–**f**). Black arrow in image *a* shows a degranulated plasmocyte, while blue arrows in images (**b**) and (**c**) mark macrophages in the interstitium. In images (**d**) and (**e**) one can see also non-granulated mast cells (black arrows). Lymphocytes are marked in image (**f**) by green arrow. All sections are stained by hematoxylin and eosin. The scale bar for all the images is 150 μm.

The histological analysis of the tissues taken from high sodium diet animals revealed several interstitial lymphocytes, degranulated mast cells, macrophages, fibroblasts as well as perivascular edema of the interstitium (see Fig. 1). In contrast to that, no macrophages were found in the tissues of the control group of animals, while the number of fibroblasts was being reduced and mast cells showed no signs of degranulation.

**Figure 2:**
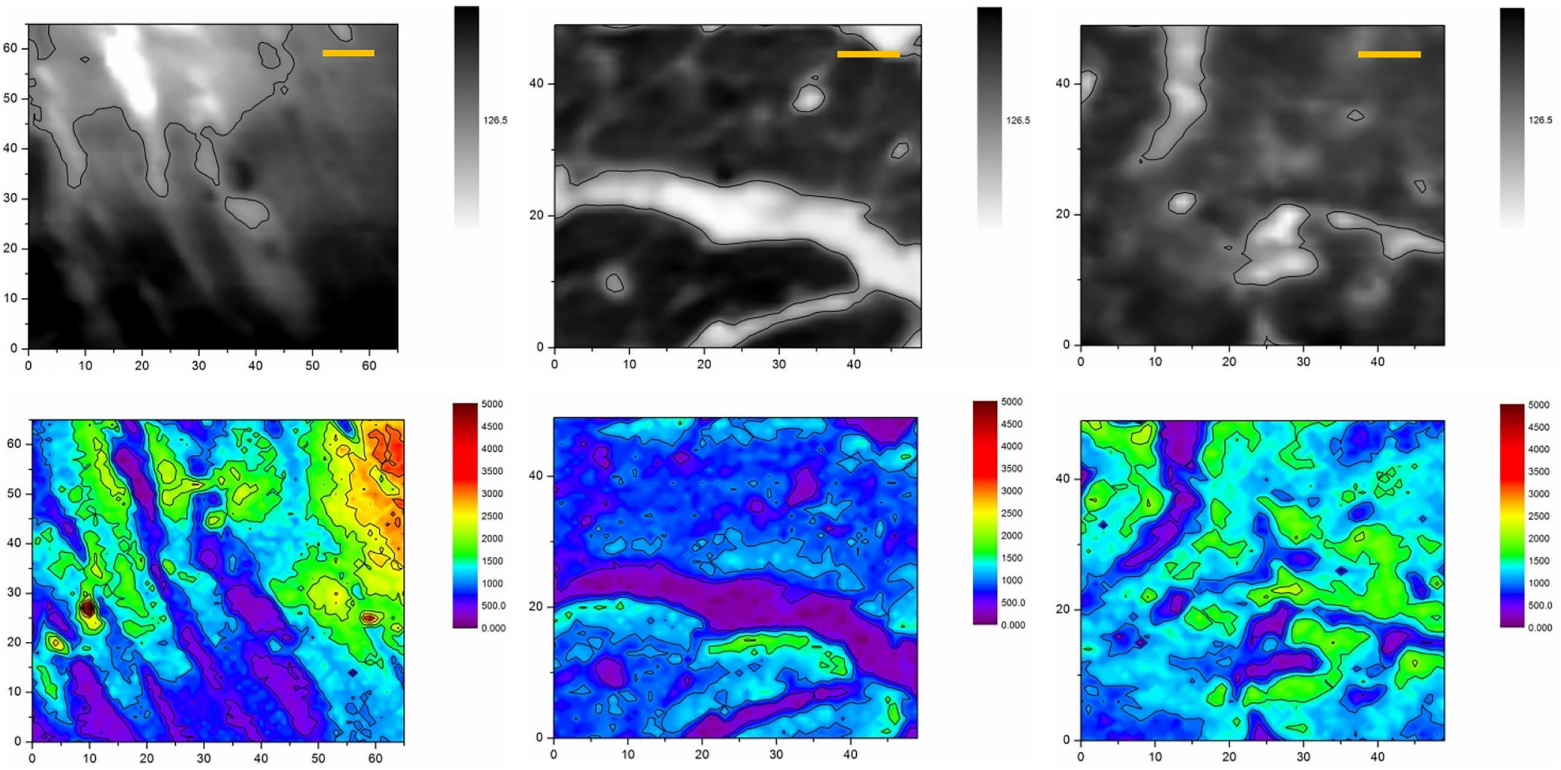
X-ray images (top row) and sodium XRF maps (bottom row) of heart muscles taken from animals of low sodium diet (control) group. Dark zones (high X-ray absorption) and light zones (low X-ray absorption) correspond to intercellular and extracellular spaces, correspondingly. The scale bars are 10 μm.

**Figure 3:**
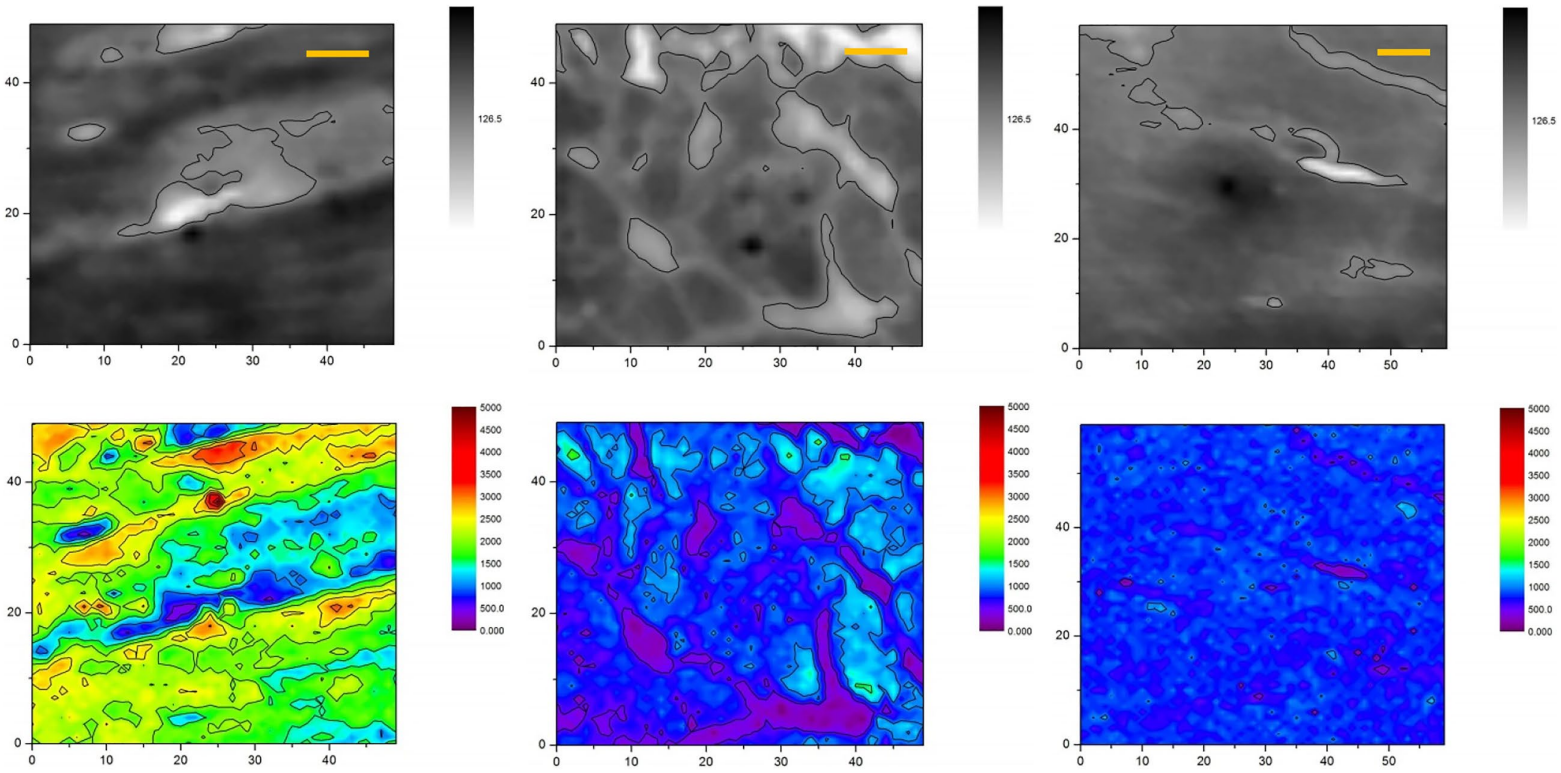
X-ray images (top row) and sodium XRF maps (bottom row) of heart muscles taken from animals of high sodium diet group. Dark zones (high X-ray absorption) and light zones (low X-ray absorption) correspond to intercellular and extracellular spaces, correspondingly. The scale bars are 10 μm.

Figures 2 and 3 show typical X-ray absorption images and corresponding sodium maps acquired with the help of synchrotron based STXM and XRF microscopy for heart tissue sections of respectively high and low sodium diet animals. In total 13 ROIs on 7 samples of rat myocardial tissues have been studied: 5 samples were extracted from 5 animals of the high sodium diet group and 2 were taken from 2 animals of the control group. The obtained STXM and XRF microscopy images enabled a clear visual segmentation of cardiomyocytes and intercellular spaces in the heart muscle tissues. In all samples, the XRF analysis revealed a higher content of the sodium content inside the cells than in the interstitium space.

This inverted correlation between intracellular and extracellular sodium can be explained by a known change of light metals content during the chemical fixation of samples. Thus, a side-by-side comparison of XRF elemental maps of cells subjected to different dehydration and drying procedures has demonstrated a significantly reduced amount of highly diffusible elements like *K, Br, Na,* and *Cl* after aldehyde-based conventional fixation^17–19^. A similar disruption of their chemical integrity in chemically fixed or poorly cryofixed cells was observed in secondary ion mass spectrometry (SIMS) experiments^20^. It should be noted that the mentioned problem has been successfully resolved by using methods of fast cryo-fixation.

One can expect that osmotically active fluid sodium would be removed from interstitium and, to a lesser extent, from intracellular volumes restricted by cell membranes. However, in our particular experiments, a wash-out of fluid sodium during the chemical fixation procedure plays a positive role: it reduces significantly a high background level of osmotic sodium of extracellular fluids to express tracks of bound sodium deposits. Due to the comparative character of the presented study and identical treatment of all the samples, there was no need to measure the absolute value of local sodium concentration with its extrapolation to in-vivo one.

**Figure 4:**
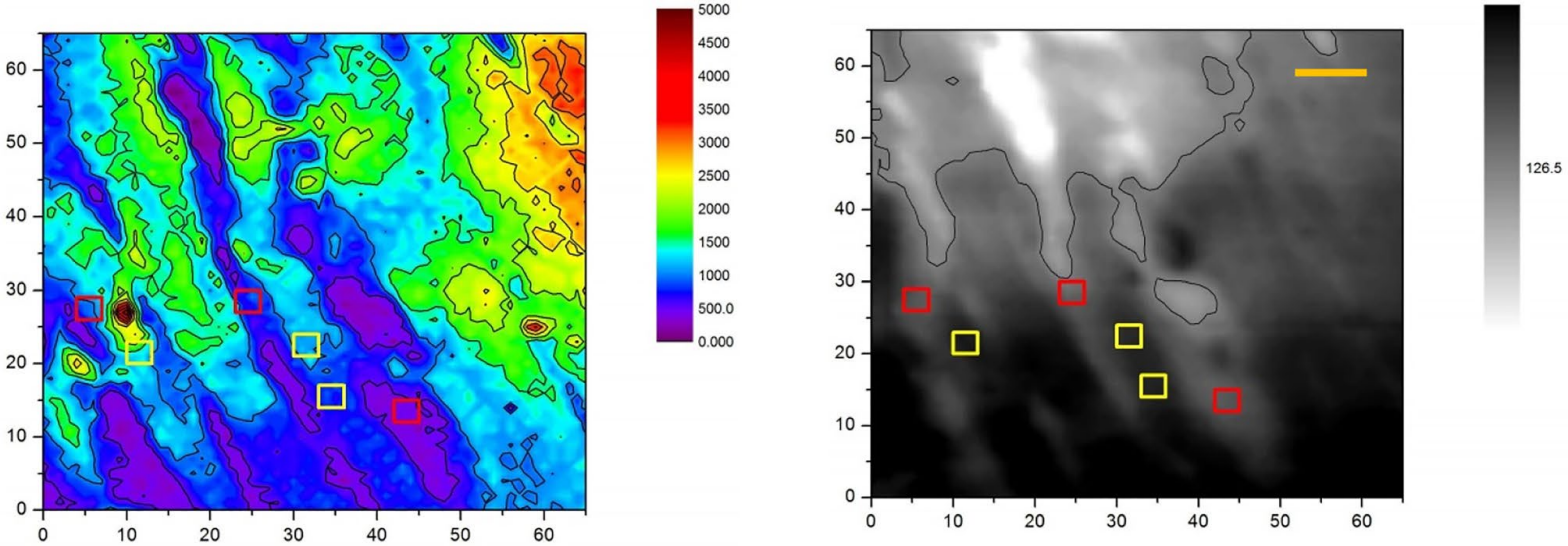
Illustration of the data sampling procedure based on combined XRF (left) and SXTM (right) images: 3 × 3 pixels zones were selected in extracellular (red squares) and intercellular (yellow squares) spaces. The scale bar is 10 μm.

One can see that sodium is accumulated mostly inside the cells with low traces in interstitium but the extracellular spaces of high sodium diet animals are filled with noticeably more sodium. To estimate this effect quantitatively we applied Kruskal–Wallis test based statistical analysis to the data obtained by XRF microscopy. The main challenge in the statistical processing was to perform an accurate data sampling inside and outside boundaries of cardiomyocytes in the context of thin slice 3D-to-2D transformation of muscle fibers and possible interstitium perforation. This problem has been solved by manual sampling of six 3 × 3 pixels (3.6 × 3.6 µm^2^) zones in every ROI to present 3 values of intracellular and 3 values of extracellular sodium content (see Fig. 4), thus forming a data sample of sodium K-line emission counts and their averaged intracellular and extracellular values for the particular ROI (see Fig. 4 and Table 1).

**Table 1:** Averaged sodium XRF counts measured in 3 intracellular and 3 extracellular points inside ROIs. B1, B2, and B4 mark the tissue samples extracted from high sodium diet animals, K5 and K7 marks the samples extracted from the control group (low sodium diet).

	Sample	ROI #	Extracellular Na			Intracellular Na		
High sodium diet	B1	1	742	834	834	1115	932	1076
		2	438	737	1097	1736	1589	1611
		3	1330	1700	1517	1823	2256	2139
	B2	1	304	367	408	574	629	624
		2	945	361	517	758	823	915
	B4	1	951	645	1041	1034	1164	1015
		2	577	413	203	1058	980	967
		3	602	193	476	969	857	603
Low sodium diet	K5	1	306	434	527	466	717	498
		2	910	501	975	1579	1469	1460
	K7	1	386	1083	649	917	1344	697
		2	261	238	194	1084	836	962
		3	302	295	387	668	694	540

As mentioned above, due to the chemical treatment used for the sample preparation, the amount of sodium inside the cells of all the samples and animals was found to be always higher than in interstitium: 1055.7 ± 445.4 against 632.8 ± 373.6 (mean value, p = 0.000012).

One can suggest that in the case of the absence of sodium deposition bound to GAG structures in the interstitium we should detect nearly the same amount of residual sodium traces in all tissues, which is defined by homeostasis control and identical sample preparation and treatment method used. However, the averaged total content of sodium (meaning no separation into intra- and extracellular spaces) measured in heart tissues for animals fed with high sodium intake rate exceeds that of control animals: 926.6 ± 483.4 against 712.6 ± 394.0 (mean values, *p* = 0.044).

**Figure 5:**
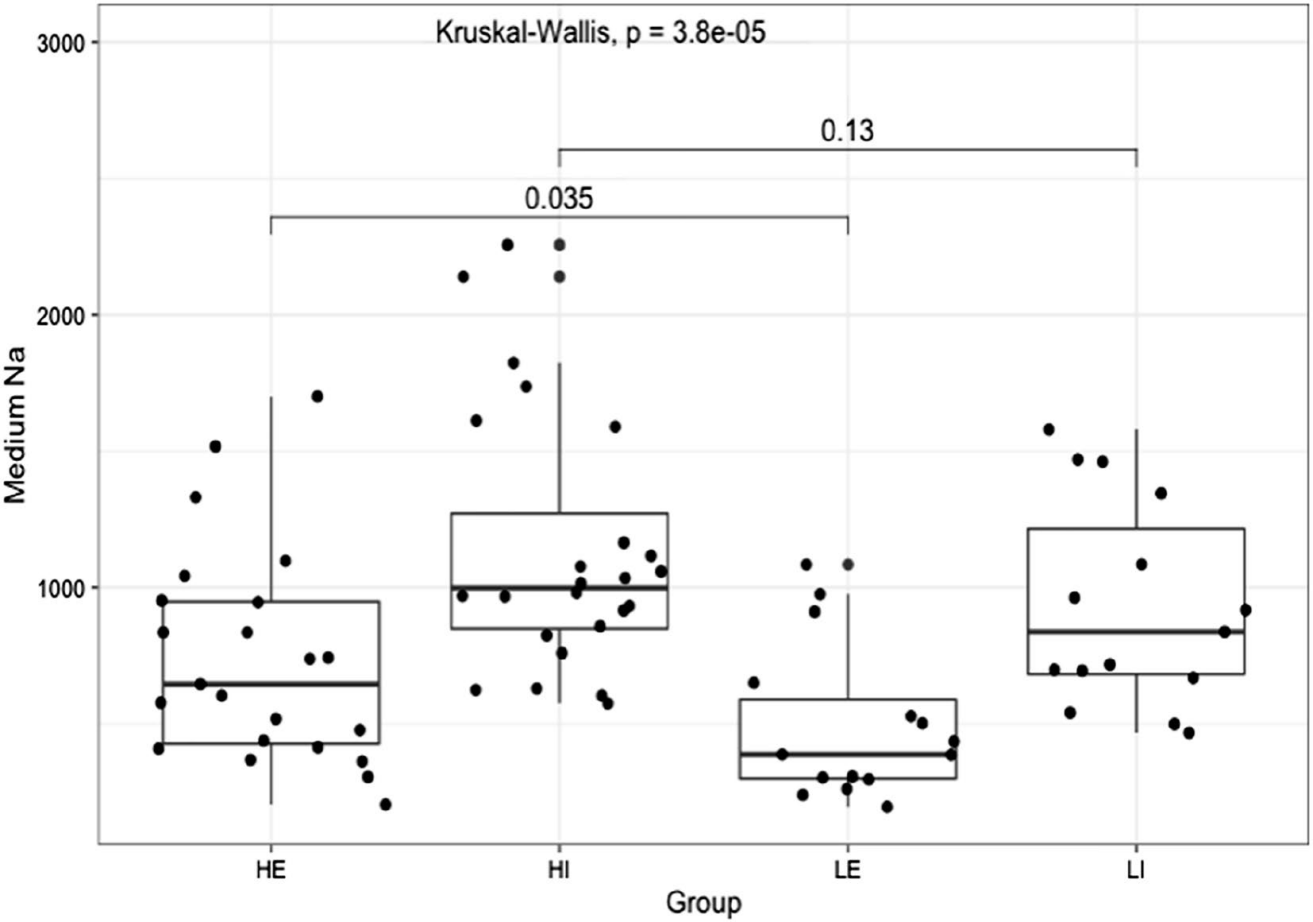
Box plot of sodium content measured in intracellular and extracellular spaces of heart tissues taken from animals fed with different rates of sodium intake. HE (High Extracellular) and HI (High Intracellular) designate data groups obtained for extracellular and intracellular contents of sodium of high sodium intake diet rats, LE (Low Extracellular) and LI (Low Intracellular) of low sodium diet rats.

The results of the statistical processing on the measured data are summarized in Fig. 5 where we present a box plot of the sodium contents measured in intracellular and extracellular spaces of heart tissues taken from different groups of animals. One can see no significant difference in the amounts of sodium content inside the cells of both high sodium and low sodium diet groups: 1135.2 ± 474.0 and 928.6 ± 375.7, correspondingly (mean values, *p* = 0.13). However, increased salt intakes result in a clearly elevated level of sodium content left in interstitium outside cardiomyocytes: 718.0 ± 402.4 for high sodium diet in comparison of 496.6 ± 283.5 for low sodium diet (mean values. *p* = 0.037, one low reliable outlier discarded).

## Discussion

Earlier investigators have studied sodium stored in tissues of biological models and humans by using flame photometry or MRI, with the MRI visualizing an overall elevated content of the tissue or organ. So far, no high spatial resolution imaging of the sodium accumulations at the cell level has been provided. Our study was aimed at more accurate localization of sodium stores in interstitium spaces of myocardial tissues of rats fed with a high rate salt diet without additional hormonal stimulation of the sodium intake.

The present investigation appeared to be successful owing to the high spectral and spatial resolution of synchrotron-based XRF microscopy. Thanks to the imaging capabilities of TwinMic microscope, an increase of sodium deposition was identified inside interstitium spaces of myocardium taken from animals with elevated levels of sodium intake. The main problem here was to recognize full and partial tissue perforations as well as different types of 2D-projections of both cardiomyocytes and interstitium volumes. Indeed, the promotion of good quality statistics also needs more strengthening by the processing of more samples and animals. The use of complimentary microtomography or XRF tomography would be profitable as well.

Nevertheless, our findings indicate elevated sodium content inside interstitium of myocardia of high sodium diet animals as compared to animals of the control group with low sodium diet. Sodium washing out of the chemical fixation removed soluble sodium from extracellular volumes, while more conservative sodium content inside cardiomyocyte membranes served as a good reference point for validation of identical parameters and treatment conditions of heart muscle slices.

In general, our results are the next step in the understanding of the relationship between stores of osmotically inactive sodium accumulated in human myocardial tissues due to excessive consumption of salt and the emergence of such clinical conditions as left ventricular hypertrophy of hypertensive patients.

## Conclusion

To our knowledge, the world's first high resolution X-ray imaging of myocardial cells by means of transmission and fluorescence X-ray microscopy techniques regarding sodium deposits has been demonstrated. The results show the elevated content of sodium in the interstitium of myocardia that can be explained by its “three-compartment” depositing and confirm the original assumption on the existence of osmotically inactive sodium allegedly bound with negatively charged glycosaminoglycans inside the interstitium network.

We believe the reported experimental results are a valuable basis to continue the research of clinically important deposition of sodium in the myocardia. The next step can be combined research and visualization of sodium and GAG structures by using a combination of immunohistochemical methods, XRF and FTIR-spectromicroscopy to validate glycosaminoglycan-sodium correlation. A cryo-fixing to conserve the chemical composition of the samples is also very promising option for future research.

## Materials and methods

### Sample preparation and animals handling

The experiments with animals were carried out in accordance with the international rules of the Guide for the Care and Use of Laboratory Animals and were approved by the Laboratory Animal Care and Use Commission and Local Ethics Committee at Pirogov Russian National Research Medical University.

Fifteen male Wistar rats aged 15–16 weeks were divided into two experimental groups possessing similar body weights: 297.4 ± 68.4 g (low sodium diet) and 252.0 ± 67.4 g (high sodium diet). The sodium consumption rate for these two groups was different starting from the third week. A low sodium diet limited the sodium intake by the value of 0.2 mEq per 200 g of body weight per day, high sodium diet ration contained 2.0 mEq or more per 200 g of body weight per day that can be considered excessive salt consumption^21,22^. The water supply was always ad libitum (25 ml of de-ionized water for 15 g of food) for all the animals, while the normal food ration being 15 g per 200 g body weight per week.

High sodium diet feeding was maintained in the corresponding group of animals during 8 weeks before the myocardial hypertrophy occurred. After 8 weeks the animals were intraperitoneally injected with methohexital at a dose of 100 mg/kg and sacrificed through decapitation.

We abide by all appropriate animal care guidelines including ARRIVE 2.0 guidelines for reporting animal research. Rats were housed in cages with a 12:12 light:dark cycle and had access to food and water ad lib at all times unless indicated otherwise. Every effort was made to ameliorate animal suffering.

### Histological staining and study

The 3-µm thick slices of the paraffin-fixed myocardia were mounted onto slides and dried (T = 60 °C) in the air. Paraffin was removed with ethanol and the slices were stained by hematoxylin and eosine in accordance with its standard procedures. The histological studies were performed with using of Leica microscope (Leica Microsystems DM2500).

### X-ray fluorescence microscopy

To investigate the sodium distributions inside and outside myocardial cells we employed X-ray fluorescence (XRF) microanalysis combined with scanning X-ray absorption microscopy (SXRM) at ELETTRA TwinMic beamline^23^, which synchrotron ring operated at the electron energy of 2.4 GeV with 160 mA current. The incident X-ray beam energy was 1.472 keV to ensure the best excitation and detection of the Kα lines of Na atoms at E = 1.041 keV.

The TwinMic microscope was operated in scanning mode. A pinhole of 75 µm diameter served as the secondary X-ray source, which was focused into 1.2 μm spot by a zone plate. The size of the probing X-ray beam was chosen as a compromise between spatial resolution, duration of the scan run and dimensions of the region of interests (ROI). The X-ray flux through the pinhole was about 3 × 10^9^ photons/s. Absorption contrast XRM images were produced with the help of a fast readout CCD camera (Andor Technology)^24^, while the simultaneous XRF signal was collected by up to eight silicon drift detectors. The exposure time was 5 s and seven detectors were used for XRF registration. Each detector had a solid acceptance angle of 3 × 10^–2^^25^.

For XRF/SXRM measurements the unstained 3-µm thick paraffin-fixed slices were put on thin Ultralene film. Neither special treatment nor coloring were applied to the samples after the chemical fixing. The TwinMic configuration enabled visualization of the myocardial tissues in respect to the corresponding sodium, magnesium and other elements distributions with precise overlapping of both the morphological and chemical data. The image size varied from 50 × 50 to 72 × 72 pixels. In total 13 ROIs selected in seven samples (3 ROIs per sample in all samples except two) of myocardial tissues have been studied using the combined XRF/SXRM microscopy. In addition to fluorescence images, the corresponding SXRM images of cardiomyocytes and interstitial space were produced.

The obtained XRF maps of chemical elements were analyzed by using open source PyMCA ver. 5.5.3 software (https://sourceforge.net/projects/pymca/files/pymca/PyMca5.5.3/)^26^.

### Statistical processing methods

For statistical processing of the data we used R language in the *RStudio* software environment (readxl, psych ggplot2^27^, ggpubr, dplyr and tidyr). The check for normality of distribution was made using the Shapiro–Wilk criteria and distribution graphs and qq-plot. The methods of nonparametric and parametric statistics were applied to calculate the quantitative indicators as mean values, standard deviations and medians (25 and 75 percentiles). The differences between the data clusters were checked by the Kruskal–Wallis and Mann–Whitney tests. On testing statistical hypotheses we rejected the null hypothesis with a significance level less than 0.05.
