# Author Correction: The first observation of osmotically neutral sodium accumulation in the myocardial interstitium

**DOI:** 10.1038/s41598-021-02956-y

**Published:** 2021-12-02

**Authors:** I. Artyukov, G. Arutyunov, M. Bobrov, I. Bukreeva, A. Cedola, D. Dragunov, R. Feshchenko, M. Fratini, V. Mitrokhin, A. Sokolova, A. Vinogradov, A. Gianoncelli

**Affiliations:** 1 P.N.Lebedev Physical Institute RAS, 53 Leninsky Prospekt, Moscow, 119991 Russia; 2 Pirogov Russian National Research Medical University, 1 Ostrovitianov St., Moscow, 117997 Russia; 3 Moscow Regional Research and Clinical Institute (MONIKI), 61/2 Shchepkina St., Moscow, 129110 Russia; 4 CNR-Institute of Nanotechnology, 5 Piazzale Aldo Moro, 00185 Rome, Italy; 5 Elettra-Sincrotrone Trieste, S.S. 14, km 163.5 in Area Science Park, 34149 Basovizza-Trieste, Italy

Correction to: *Scientific Reports* 10.1038/s41598-021-01443-8, published online 11 November 2021

The original version of this Article contained an error in the Acknowledgments section.

“Part of the research reported in this publication was also supported in the frames of HORIZON 2020 Program.”

now reads:

“The research leading to this result has been supported by the project CALIPSOplus under Grant Agreement 730872 from the EU Framework Programme for Research and Innovation HORIZON 2020.”

The original Article has been corrected.

